# Childhood body mass index trajectories and associations with adult-onset chronic kidney disease in Denmark: A population-based cohort study

**DOI:** 10.1371/journal.pmed.1004098

**Published:** 2022-09-21

**Authors:** Julie Aarestrup, Kim Blond, Dorte Vistisen, Marit E. Jørgensen, Marie Frimodt-Møller, Britt W. Jensen, Jennifer L. Baker

**Affiliations:** 1 Center for Clinical Research and Prevention, Copenhagen University Hospital—Bispebjerg and Frederiksberg, Frederiksberg, Denmark; 2 Steno Diabetes Center Copenhagen, Herlev, Denmark; 3 Department of Public Health, University of Copenhagen, Copenhagen K, Denmark; 4 Steno Diabetes Center Greenland, Nuuk, Greenland; 5 National Institute of Public Health, University of Southern Denmark, Copenhagen, Denmark; Royal Derby Hospital, UNITED KINGDOM

## Abstract

**Background:**

Although excess adult adiposity is a strong risk factor for chronic kidney disease (CKD), evidence for associations with early life body size is limited. We investigated whether childhood body mass index (BMI) trajectories are associated with adult-onset CKD and end-stage kidney disease (ESKD) using a population-based cohort. Further, we examined the role of adult-onset type 2 diabetes (T2D) in these associations.

**Methods and findings:**

We included 151,506 boys and 148,590 girls from the Copenhagen School Health Records Register, born 1930 to 1987 with information on measured weights and heights at ages 6 to 15 years. Five sex-specific childhood BMI trajectories were analyzed. Information on the main outcomes CKD and ESKD, as well as T2D, came from national health registers. Incidence rate ratios (IRRs) and 95% confidence intervals (CIs) were estimated using Poisson regression adjusted for year of birth. During a median of 30.8 person-years of follow-up, 5,968 men and 3,903 women developed CKD and 977 men and 543 women developed ESKD. For both sexes, the rates of CKD and ESKD increased significantly with higher child BMI trajectories in comparison with the average BMI trajectory (40% to 43% of individuals) and the below-average BMI trajectory (21% to 23% of individuals) had the lowest rates. When including T2D, most associations were significant and men (IRR = 1.39, 95% CI: 1.13 to 1.72) and women (IRR = 1.54, 95% CI: 1.28 to 1.86) with the obese childhood BMI trajectory (2% of individuals) had significantly higher CKD rates than the average BMI trajectory, whereas for ESKD, the associations were positive, but nonsignificant, for men (IRR = 1.38, 95% CI: 0.83 to 2.31) but significant for women (IRR = 1.97, 95% CI: 1.25 to 3.11) with the obese BMI trajectory. A main study limitation is the use of only hospital-based CKD diagnoses.

**Conclusions:**

Individuals with childhood BMI trajectories above average had higher rates of CKD and ESKD than those with an average childhood BMI trajectory. When including T2D, most associations were significant, particularly with CKD, emphasizing the potential information that the early appearance of above-average BMI growth patterns provide in relation to adult-onset CKD beyond the information provided by T2D development.

## Abstract 2

Using a population-based cohort, Julie Aarestrup and colleagues investigate whether childhood body mass index trajectories are associated with adult-onset chronic kidney disease and end-stage kidney disease.

## Author summary

**Why was this study done?:**

- Worldwide, chronic kidney disease (CKD) is common and associated with numerous adverse health effects.
- Severe obesity in childhood is associated with impaired kidney health.
- Little is known on the impact of childhood body size on subsequent development of CKD.

**What did the researchers do and find?:**

- Using a large Danish population-based cohort of 151,506 boys and 148,590 girls, we investigated if trajectories of childhood body mass index (BMI) development were associated with adult-onset CKD.
- In analyses that include type 2 diabetes, men with above-average (incidence rate ratio [IRR] = 1.07, 95% confidence interval [CI]: 1.00 to 1.14), overweight (IRR = 1.25, 95% CI: 1.14 to 1.38), and obese (IRR = 1.39, 95% CI: 1.13 to 1.72) childhood BMI trajectories had higher rates of adult-onset CKD compared to those with an average childhood BMI trajectory as did women with above-average (IRR = 1.18, 95% CI: 1.08 to 1.28), overweight (IRR = 1.24, 95% CI: 1.11 to 1.38), and obese (IRR = 1.54, 95% CI: 1.28 to 1.86) childhood BMI trajectories.

**What do these findings mean?:**

- Our findings highlight the importance of childhood BMI development in the identification of individuals at risk of adult-onset CKD.
- Our study was limited by only including the more severe CKD cases.
- The results suggest that the high worldwide prevalence of childhood overweight and obesity may contribute to the future burden of CKD.
- Furthermore, these findings open up new avenues for exploration into the mechanisms that increase the risk of this common health outcome.

## Introduction

The burden of chronic kidney disease (CKD) is rising worldwide due to population aging and an increased prevalence of CKD risk factors [1]. An estimated 697.5 million individuals, corresponding to a global prevalence of 9.1%, had a diagnosis of CKD in 2017 [1]. Further, 1.2 million individuals died from CKD in 2017 and the disease is projected to rise in rank as a leading cause of death [1]. CKD is generally more common in women than men; however, the disease is often more severe among men as indicated by the higher rates of end-stage kidney disease (ESKD) and age-standardized mortality [1]. Although CKD is recognized as an important health issue, the awareness of the disease remains low among the general population and healthcare providers [2].

CKD is defined by kidney damage and includes a range of abnormalities in kidney structure or function [3]. The disorder encompasses different disease stages, of which some early stages can be asymptomatic in contrast to the clinically apparent advanced-stages, including ESKD. The disease is associated with a range of adverse effects, including cardiovascular complications, reduced quality of life, and premature mortality [2].

The primary risk factors for CKD vary by country; however, diabetes and hypertension are among the established leading causes, particularly in high-income countries [2,4]. Additionally, increasing evidence supports that excess adult adiposity is a substantial and causal contributor to CKD risk [5–8]. Although overweight and obesity are closely related to both diabetes and hypertension, it is suggested that excess adiposity may have effects on CKD risks independent of these 2 major causes of CKD [9].

In addition to the high prevalence of overweight in adults, overweight and obesity in children now similarly constitute an enormous global health crisis with more than 124 million children living with obesity [10]. Despite the presence of early kidney abnormalities and decreased kidney function among children with severe obesity [7], the potential early origins of CKD are sparsely investigated [11–16]. Among the few studies in this area, some included body size in late adolescence [12,14], and many only provided associations with ESKD [11,12,14]. Further, although some studies investigated associations with patterns of BMI development from child- to adulthood [13,15,16], the majority of these were limited by including subclinical renal damage as a proxy for CKD [15,16]. Therefore, using a large Danish cohort with repeated measurements of childhood height and weight, we prospectively investigated sex-specific associations between childhood BMI trajectories and weight status at individual childhood ages and adult-onset CKD and ESKD, respectively, and the role of adult-onset type 2 diabetes (T2D) on these associations.

## Methods

### Cohort

Children included in this population-based cohort study came from the Copenhagen School Health Records Register (CSHRR), which includes computerized information on virtually all children born 1930 to 1996, who attended a private or public school in Copenhagen, Denmark [17]. Currently, 406,350 children (200,977 girls) are included in the register. Trained school physicians or nurses performed the health examinations using standardized procedures during the school years. Height and weight measurements are available at ages 6 to 15 years, allowing for the calculation of BMI (kg/m^2^). Height was measured without shoes and weight was measured either naked or in underwear only. After the 1970s, light clothing was permitted [17].

### Childhood latent class trajectories and weight status

The children in the CSHRR had up to 12 measurements recorded. All children with a minimum of 2 BMI values (≥95% of children had more than 2 values) were included when estimating sex-specific BMI trajectories using latent class trajectory models, which impute missing values under a missing at random assumption [18–20]. We a priori chose to generate BMI trajectories separately by sex to account for differences in growth among boys and girls. The trajectories were modelled using natural splines with knot points positioned at ages 8, 10, and 12 years (corresponding approximately to the 25th, 50th, and 75th percentiles) to best capture the natural growth patterns of children at these ages as well as to evenly space these points across the age span and were adjusted for birth cohort (5-year intervals). Several parameters and fit indices (i.e., proportion in each trajectory, mean posterior probability, Bayesian information criteria, relative entropy, odds of correct classification, visual inspection of the trajectories) were used to identify the optimal number of trajectories [19]. We tested models with up to 8 different childhood BMI trajectories (S1 and S2 Figs). The best performing model identified 5 noncrossing BMI trajectories for each sex (S3 Fig) and did not include random effects, whereby the variation in BMI level within each trajectory was reduced. These BMI trajectories were termed as below-average, average, above-average, overweight, and obese. Most boys and girls followed the average BMI trajectory, and the fewest belonged to the obese BMI trajectory (Table 1). For each child, a posterior probability, which is a measure of how well a child’s BMI trajectory fits within the identified trajectories, was assigned for each of the 5 trajectories. Furthermore, due to nonlinearity in the associations, at individual childhood ages, BMI values were classified as underweight (<fifth BMI percentile), normal weight (≥fifth and <85th BMI percentile), or overweight, including obesity (≥85th BMI percentile) using the Centers for Disease Control and Prevention (CDC) criteria [21].

**Table 1:** Characteristics of individuals in the CSHRR by childhood BMI trajectory and by sex.

			BMI trajectory*				
Sex	Characteristic	All (n)	Below-average	Average	Above-average	Overweight	Obese
Men	n (%)	151,506	34,945 (23.1)	64,548 (42.6)	37,721 (24.9)	11,854 (7.8)	2,438 (1.6)
	Year of birth, median (IQR)	151,506	1950 (1941–1963)	1948 (1940–1962)	1949 (1941–1963)	1954 (1944–1970)	1965 (1950–1980)
	BMI at age 7 years, median (IQR)	142,561	14.3 (13.9–14.7)	15.3 (14.9–15.7)	16.2 (15.7–16.7)	17.2 (16.5–17.9)	19.1 (18.0–20.3)
	BMI at age 10 years, median (IQR)	137,454	15.0 (14.5–15.3)	16.2 (15.8–16.6)	17.6 (17.1–18.1)	19.6 (18.9–20.4)	22.7 (21.7–24.1)
	BMI at age 13 years, median (IQR)	132,157	16.0 (15.4–16.4)	17.5 (17.0–18.1)	19.4 (18.9–20.1)	22.0 (21.2–23.0)	25.8 (24.7–27.3)
	CKD, n (%)	5,968 (3.9)	1,198 (3.4)	2,554 (4.0)	1,583 (4.2)	539 (4.5)	94 (3.9)
	ESKD, n (%)	977 (0.6)	198 (0.6)	431 (0.7)	253 (0.7)	79 (0.7)	16 (0.7)
	T2D, n (%)	18,004 (11.9)	3,606 (10.3)	7,226 (11.2)	4,787 (12.7)	1,945 (16.4)	440 (18.0)
	Death, n (%)	38,241 (25.2)	8,755 (25.1)	17,082 (26.5)	9,585 (25.4)	2,431 (20.5)	388 (15.9)
Women	n (%)	148,590	31,027 (20.9)	59,224 (39.9)	39,823 (26.8)	15,057 (10.1)	3,459 (2.3)
	Year of birth, median (IQR)	148,590	1950 (1942–1963)	1948 (1940–1962)	1949 (1940–1962)	1952 (1943–1967)	1960 (1947–1977)
	BMI at age 7 years, median (IQR)	139,670	14.0 (13.6–14.5)	15.0 (14.6–15.5)	16.0 (15.4–16.6)	17.2 (16.5–18.0)	19.1 (18.1–20.3)
	BMI at age 10 years, median (IQR)	135,381	14.7 (14.2–15.1)	16.0 (15.6–16.5)	17.5 (17.0–18.2)	19.6 (18.8–20.4)	22.5 (21.5–23.7)
	BMI at age 13 years, median (IQR)	131,377	16.0 (15.4–16.5)	17.8 (17.3–18.4)	19.9 (19.2–20.6)	22.4 (21.6–23.3)	26.1 (24.9–27.5)
	CKD, n (%)	3,903 (2.6)	662 (2.1)	1,467 (2.5)	1,195 (3.0)	452 (3.0)	127 (3.7)
	ESKD, n (%)	543 (0.4)	74 (0.2)	202 (0.3)	169 (0.4)	76 (0.5)	22 (0.6)
	T2D, n (%)	12,427 (8.4)	2,022 (6.5)	4,318 (7.3)	3,603 (9.0)	1,875 (12.5)	609 (17.6)
	Death, n (%)	27,816 (18.7)	5,356 (17.3)	11,312 (19.1)	7,890 (19.8)	2,752 (18.3)	506 (14.6)

BMI, body mass index; CKD, chronic kidney disease; CSHRR, Copenhagen School Health Records Register; ESKD, end-stage kidney disease; IQR, interquartile range; T2D, type 2 diabetes.

*For descriptive purposes, individuals were assigned to the trajectory with the highest posterior probability.

### Linkages and case ascertainment

In Denmark, all citizens alive or born after April 2, 1968 were issued a unique government identification number [22]. These numbers were recorded on the children’s health cards or retrieved for children who finished school before 1968 [17]. Through these numbers, individual-level linkage of children in the CSHRR to the Danish Vital Statistics Register for vital status information was performed [22]. Information on adult-onset CKD, including ESKD, was obtained from the Danish National Patient Register, which contains information on discharge diagnoses from all inpatient hospital contacts since 1977 and all outpatient hospital contacts since 1995 [23]. CKD was defined based upon the first hospital admission or contact using the International Classification of Disease eight revision until 1994 and the 10th revision thereafter (S1 Table). Information on T2D diagnoses came from several national health registers, and information on kidney cancer was retrieved through linkage to the Danish Cancer Registry, where information is available from 1943 onwards (S1 Table) [24].

### Study population and follow-up

Individuals eligible for inclusion into this study were born 1930 to 1987, had an identification number, and were alive and living in Denmark at age 30 years or older from January 1, 1977 onwards (*n* = 311,873) (S4 Fig). Exclusions were made for individuals with a diagnosis of CKD (inclusive of hereditary kidney disease) or kidney cancer (*n* = 507) or diabetes (*n* = 1,007) before follow-up (i.e., before age 30 years or before 1977), and those with <2 childhood BMI values (*n* = 10,263). The analytical population consisted of 300,096 individuals (151,506 men, 148,590 women). Follow-up started on January 1, 1977 or at age 30 years, whichever came later, and ended on the date of a diagnosis of CKD or ESKD, hereditary kidney disease, kidney cancer, type 1 diabetes, death, emigration, loss to follow-up, or December 31, 2017, whichever came first. We further censored individuals if they had a first diagnosis of T2D and CKD on the same date.

### Statistical analyses

For descriptive statistics, a child was assigned to the BMI trajectory with the highest posterior probability. Medians and interquartile ranges (IQRs) are presented by childhood BMI trajectory and by sex.

A priori, we chose to estimate sex-specific incidence rate ratios (IRRs) and 95% confidence intervals (CIs) of the associations between childhood body size exposures and adult-onset CKD and ESKD using Poisson regression analyses. The logarithm of time at risk was used as the offset and age (in 1-year time intervals to accommodate that the rates of the outcomes changes with age) and birth year were included as covariates. In analyses on childhood BMI trajectories, posterior probabilities, which have been found to be superior to modal assignment [25], were used as the exposures to reduce potential effects of misclassification among the trajectories. The average BMI trajectory was used as the reference, and the posterior probabilities were included as 4 continuous variables, leaving out the reference. To further enhance comparability with other studies, we also performed analyses with childhood BMI categorized as under, normal, and overweight (including obesity) at ages 7, 10, and 13 years. We examined potential interactions of birth cohort (modelled using 3 categories: 1930 to 1939, 1940 to 1949, and 1950 to 1987) on the associations between the childhood BMI trajectories (S2 Table) and childhood weight status (S3 Table), respectively, and CKD using the likelihood ratio test. We did not detect any interactions on associations with CKD (*p*-values ≥ 0.06), although there were indications that the associations strengthened in magnitude across time in men, but not women, with childhood BMI trajectories above average and with childhood overweight at individual ages, respectively. Due to a low case number, we were not sufficiently powered to conduct similar investigations on associations with ESKD.

An examination of potential effect modification by T2D on the associations between the childhood body size exposures and outcomes was planned a priori. We included a product term between T2D and the posterior probabilities for childhood BMI trajectory membership and childhood weight status at specific ages, respectively, and tested for potential interactions using the likelihood ratio test, while allowing for an interaction between T2D and age at risk. Further, we conducted analyses that did and did not include T2D modelled as a time-varying covariate to account for the duration of T2D to evaluate if childhood body size provides information about adult-onset CKD and ESKD rates beyond that from T2D.

Sex-specific 10-year cumulative incidences of CKD and ESKD were planned a priori and estimated at ages 55 and 65 years in individuals with and without T2D, respectively, by childhood BMI trajectories using the conditional survival function, taking competing risk of death into account [26]. The related 95% CIs were simulated using the bootstrap method with 1,000 repetitions [27].

This study is reported as per the Strengthening the Reporting of Observational Studies in Epidemiology (STROBE) guideline (S1 STROBE Checklist).

### Statement of ethics

This study was approved by the Danish Data Protection Agency (Datatilsynet), approval number 2012-58-0004. According to Danish law, ethical approval or informed consent are not required for register-based studies.

## Results

During a median of 30.8 (IQR: 18.7 to 40.0) person-years of follow-up, 5,968 men and 3,903 women were diagnosed with CKD and 977 men and 543 women with ESKD (Table 1). The median age at diagnosis for CKD was 67 years (IQR: 57 to 73 years) for men and 66 years (IQR: 55 to 74 years) for women and for ESKD the median age at diagnosis was 62 years (IQR: 51 to 71 years) for men and 62 years (IQR: 50 to 70 years) for women.

**Fig 1:**
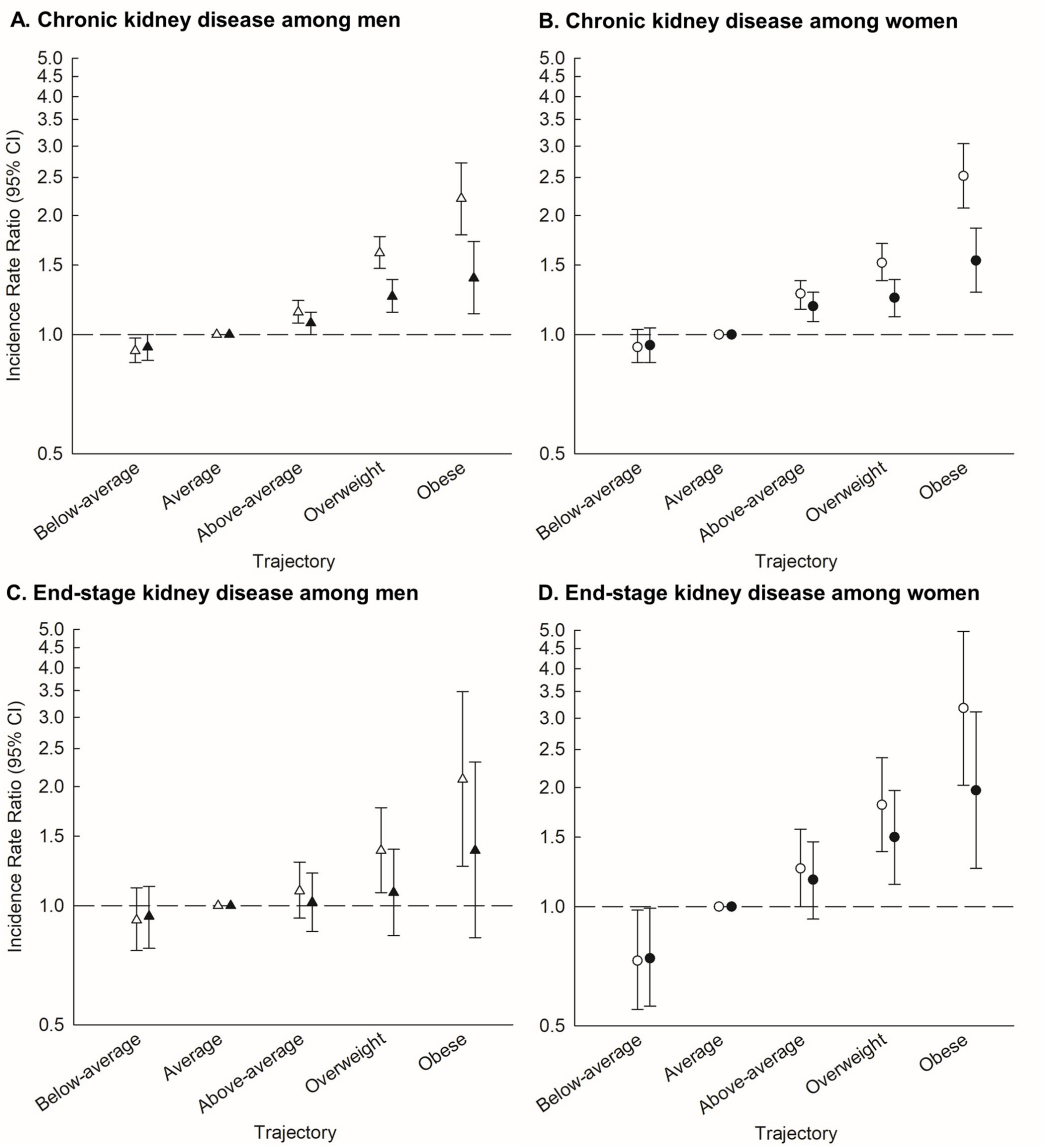
Childhood BMI trajectories and CKD and ESKD. (A) CKD among men in analyses without (white) and with (black) T2D. (B) CKD among women in analyses without (white) and with (black) T2D. (C) ESKD among men in analyses without (white) and with (black) T2D. (D) ESKD among women in analyses without (white) and with (black) T2D. BMI, body mass index; CI, confidence interval; CKD, chronic kidney disease; ESKD, end-stage kidney disease; T2D, type 2 diabetes.

A significantly lower rate of CKD was observed for men with the below-average childhood BMI trajectory in comparison with the average trajectory (used as the reference) (Fig 1A and S4 Table). In contrast, men with the above-average BMI, overweight, and obese childhood BMI trajectories had significantly higher rates of CKD, in comparison with the average BMI reference group, and the strength of the association increased in a dose–response manner. The patterns of associations with rates of CKD were similar among women. Women with childhood BMI trajectories that were above-average, overweight, and obese had significantly higher CKD rates than those with the average childhood BMI trajectory (Fig 1B and S4 Table). Although the association with the below-average childhood BMI trajectory did not reach statistical significance among women, it still indicated a lower CKD rate. We did not find that the occurrence of adult-onset T2D significantly modified the associations between childhood BMI trajectories and rates of CKD among men (*p*-value = 0.19) or women (*p*-value = 0.90). When including T2D in the analyses, the associations were statistically significant among men with the above-average (IRR = 1.07, 95% CI: 1.00 to 1.14), overweight (IRR = 1.25, 95% CI: 1.14 to 1.38), and obese (IRR = 1.39, 95% CI: 1.13 to 1.72) childhood BMI trajectories and women with the above-average (IRR = 1.18, 95% CI: 1.08 to 1.28), overweight (IRR = 1.24, 95% CI: 1.11 to 1.38), and obese (IRR = 1.54, 95% CI: 1.28 to 1.86) childhood BMI trajectories. When including T2D, associations with the below-average childhood BMI trajectory did not reach statistical significance in men (IRR = 0.93, 95% CI: 0.86 to 1.00) or women (IRR = 0.94, 95% CI: 0.85 to 1.04).

Investigations of ESKD generally showed similar patterns of associations with childhood BMI trajectories as were found for CKD. In analyses that did not include adult-onset T2D, men with the overweight and obese childhood BMI trajectories had significantly higher rates of ESKD than men with the average childhood BMI trajectory, whereas no significant associations were observed for the below- and the above-average childhood BMI trajectories (Fig 1C and S4 Table). Among women in analyses that did not include T2D, those with the above-average, overweight, and obese childhood BMI trajectories had significantly higher ESKD rates than women with the average childhood BMI trajectory, whereas women with the below-average BMI trajectory had a significantly lower ESKD rate (Fig 1D and S2 Table). We did not find that the occurrence of adult-onset T2D significantly modified the associations between childhood BMI trajectories and rates of ESKD among men (*p*-value = 0.29) or women (*p*-value = 0.83). When including adult-onset T2D, the associations were not statistically significant among men. Among women, when including T2D, those with the overweight (IRR = 1.50, 95% CI: 1.14 to 1.97) and obese (IRR = 1.97, 95% CI: 1.25 to 3.11) childhood BMI trajectories had significantly higher rates of ESKD compared with the average childhood BMI trajectory, and those with the below-average BMI trajectory (IRR = 0.74, 95% CI: 0.56 to 0.99) had a significantly lower ESKD rate.

The cumulative incidences for CKD were highest for the childhood BMI trajectories with the highest mean BMI, at older adult ages, among men and among individuals with adult-onset T2D (Table 2). A similar pattern was observed for ESKD, although the cumulative incidences were lower than for CKD (Table 2).

**Table 2:** Cumulative incidences of CKD and ESKD by childhood BMI trajectory. Sex-specific 10-year cumulative incidences (in percentages) of CKD and ESKD in individuals aged 55 and 65 years with and without T2D, respectively, by childhood BMI trajectories when taking competing risk of death into account.

			Men				Women			
			Cumulative incidence (95% CI)				Cumulative incidence (95% CI)			
Disease	Age (years)	BMI trajectory	Without T2D		With T2D		Without T2D		With T2D	
CKD	55	Below-average	0.73	(0.69–0.79)	6.46	(5.87–7.13)	0.51	(0.47–0.56)	3.89	(3.39–4.49)
		Average	0.79	(0.75–0.83)	6.93	(6.36–7.56)	0.54	(0.51–0.58)	4.11	(3.62–4.67)
		Above-average	0.84	(0.79–0.90)	7.38	(6.73–8.04)	0.64	(0.59–0.68)	4.81	(4.24–5.43)
		Overweight	0.98	(0.90–1.08)	8.55	(7.67–9.48)	0.67	(0.60–0.74)	5.02	(4.36–5.73)
		Obese	1.09	(0.87–1.36)	9.33	(7.50–11.56)	0.83	(0.68–1.01)	6.17	(5.01–7.59)
	65	Below-average	1.79	(1.68–1.90)	7.91	(7.30–8.62)	1.07	(0.98–1.16)	5.36	(4.78–6.04)
		Average	1.92	(1.84–2.01)	8.50	(7.91–9.12)	1.13	(1.07–1.20)	5.67	(5.13–6.27)
		Above-average	2.05	(1.94–2.16)	9.03	(8.34–9.72)	1.33	(1.24–1.41)	6.60	(5.95–7.27)
		Overweight	2.39	(2.18–2.60)	10.41	(9.43–11.44)	1.38	(1.25–1.52)	6.82	(6.05–7.66)
		Obese	2.62	(2.10–3.24)	11.17	(9.01–13.74)	1.71	(1.41–2.07)	8.32	(6.81–10.10)
ESKD	55	Below-average	0.19	(0.16–0.22)	0.72	(0.56–0.95)	0.08	(0.06–0.11)	0.46	(0.31–0.69)
		Average	0.20	(0.18–0.22)	0.77	(0.61–0.98)	0.11	(0.09–0.13)	0.61	(0.43–0.86)
		Above-average	0.20	(0.18–0.23)	0.78	(0.61–0.99)	0.13	(0.11–0.15)	0.71	(0.50–0.99)
		Overweight	0.21	(0.17–0.27)	0.83	(0.61–1.10)	0.16	(0.13–0.21)	0.90	(0.63–1.28)
		Obese	0.27	(0.16–0.46)	1.04	(0.60–1.81)	0.21	(0.14–0.34)	1.19	(0.71–2.00)
	65	Below-average	0.29	(0.25–0.34)	0.60	(0.47–0.77)	0.12	(0.09–0.15)	0.36	(0.25–0.53)
		Average	0.31	(0.28–0.35)	0.64	(0.51–0.80)	0.16	(0.14–0.19)	0.48	(0.35–0.66)
		Above-average	0.32	(0.28–0.36)	0.65	(0.51–0.81)	0.18	(0.15–0.22)	0.56	(0.40–0.75)
		Overweight	0.34	(0.27–0.42)	0.69	(0.51–0.91)	0.23	(0.18–0.30)	0.70	(0.50–0.98)
		Obese	0.42	(0.25–0.72)	0.85	(0.48–1.48)	0.31	(0.19–0.49)	0.92	(0.55–1.54)

BMI, body mass index; CI, confidence interval; CKD, chronic kidney disease; ESKD, end-stage kidney disease; T2D, type 2 diabetes.

When categorizing childhood weight status at ages 7, 10, and 13 years, most boys and girls were classified as having a normal weight, which was used as the reference group, and the fewest as having underweight (Table 3). Men and women with overweight at childhood ages had higher rates of CKD in comparison with individuals with normal weight in childhood, whereas childhood underweight was generally not associated with adult-onset CKD (Table 3). We did not find strong indications of an effect modification by T2D in the analyses of childhood age-specific weight status and rates of CKD among men (*p*-values ≥ 0.39) or women (*p*-values ≥ 0.23), except for 1 significant interaction with weight status at age 13 years among boys (*p*-value = 0.03). When including adult-onset T2D in the analyses, the rates of CKD were significantly higher among men (IRR_age 13 years_ = 1.37, 95% CI: 1.24 to 1.52) and women (IRR_age 13 years_ = 1.36, 95% CI: 1.22 to 1.53) with childhood overweight in comparison to individuals with childhood normal weight and the estimates were higher with older childhood ages (Table 3). Both men and women with overweight at childhood ages had significantly higher rates of ESKD than those with normal weight, while underweight in childhood was not associated with ESKD (Table 3). We did not find indications of an effect modification by T2D in the analyses of childhood age-specific weight status and rates of ESKD among men (*p*-values ≥ 0.50) or women (*p*-values ≥ 0.43). When including adult-onset T2D in the analyses for ESKD, the associations with overweight in childhood were not statistically significant for men (IRR_age 13 years_ = 1.22, 95% CI: 0.94 to 1.58) but were statistically significant for women at ages 10 and 13 years (IRR_age 13 years_ = 1.83, 95% CI: 1.40 to 2.39).

**Table 3:** Childhood weight status and CKD and ESKD. Sex-specific IRRs and corresponding 95% CIs of the associations between childhood weight status at ages 7, 10, and 13 years and CKD and ESKD, respectively, in analyses with and without adult-onset T2D and duration with T2D.

				All	Cases	Without T2D		With T2D	
Sex	Disease	Age (years)	Weight status	n (%*)	n (%^†^)	IRR	95% CI	IRR	95% CI
Men	CKD	7	Underweight	6,593 (4.6)	245 (3.7)	1.01	(0.88–1.14)	0.98	(0.87–1.12)
			Normal weight	126,613 (88.8)	5,007 (4.0)	1 (ref)		1 (ref)	
			Overweight	9,355 (6.6)	339 (3.6)	1.33	(1.19–1.49)	1.16	(1.04–1.30)
		10	Underweight	4,246 (3.1)	152 (3.6)	1.01	(0.86–1.18)	1.04	(0.89–1.23)
			Normal weight	124,122 (90.3)	5,168 (4.2)	1 (ref)		1 (ref)	
			Overweight	9,086 (6.6)	427 (4.7)	1.69	(1.53–1.87)	1.29	(1.17–1.43)
		13	Underweight	7,753 (5.9)	267 (3.4)	0.91	(0.80–1.03)	0.97	(0.86–1.10)
			Normal weight	116,466 (88.1)	4,930 (4.2)	1 (ref)		1 (ref)	
			Overweight	7,938 (6.0)	421 (5.3)	1.92	(1.74–2.12)	1.37	(1.24–1.52)
	ESKD	7	Underweight	6,593 (4.6)	28 (0.4)	0.69	(0.47–1.01)	0.69	(0.47–1.00)
			Normal weight	126,613 (88.8)	829 (0.7)	1 (ref)		1 (ref)	
			Overweight	9,355 (6.6)	58 (0.6)	1.34	(1.03–1.75)	1.16	(0.89–1.52)
		10	Underweight	4,246 (3.1)	22 (0.5)	0.87	(0.57–1.33)	0.90	(0.59–1.38)
			Normal weight	124,122 (90.3)	858 (0.7)	1 (ref)		1 (ref)	
			Overweight	9,086 (6.6)	64 (0.7)	1.47	(1.14–1.90)	1.13	(0.87–1.46)
		13	Underweight	7,753 (5.9)	43 (0.6)	0.88	(0.65–1.19)	0.95	(0.70–1.29)
			Normal weight	116,466 (88.1)	820 (0.7)	1 (ref)		1 (ref)	
			Overweight	7,938 (6.0)	64 (0.8)	1.68	(1.30–2.16)	1.22	(0.94–1.58)
Women	CKD	7	Underweight	6,260 (4.5)	154 (2.5)	1.00	(0.85–1.18)	0.99	(0.84–1.16)
			Normal weight	123,996 (88.8)	3,201 (2.6)	1 (ref)		1 (ref)	
			Overweight	9,414 (6.7)	262 (2.8)	1.46	(1.28–1.65)	1.20	(1.06–1.36)
		10	Underweight	5,107 (3.8)	102 (2.0)	0.85	(0.69–1.03)	0.87	(0.71–1.06)
			Normal weight	121,788 (90.0)	3,364 (2.8)	1 (ref)		1 (ref)	
			Overweight	8,486 (6.3)	275 (3.2)	1.60	(1.41–1.81)	1.22	(1.07–1.38)
		13	Underweight	6,101 (4.6)	126 (2.1)	0.83	(0.69–0.99)	0.88	(0.74–1.05)
			Normal weight	116,349 (88.6)	3,237 (2.8)	1 (ref)		1 (ref)	
			Overweight	8,927 (6.8)	350 (3.9)	1.87	(1.67–2.09)	1.36	(1.22–1.53)
	ESKD	7	Underweight	6,260 (4.5)	15 (0.2)	0.70	(0.42–1.18)	0.70	(0.42–1.17)
			Normal weight	123,996 (88.8)	447 (0.4)	1 (ref)		1 (ref)	
			Overweight	9,414 (6.7)	41 (0.4)	1.65	(1.19–2.27)	1.35	(0.98–1.87)
		10	Underweight	5,107 (3.8)	12 (0.2)	0.75	(0.42–1.32)	0.77	(0.43–1.37)
			Normal weight	121,788 (90.0)	459 (0.4)	1 (ref)		1 (ref)	
			Overweight	8,486 (6.3)	50 (0.6)	2.15	(1.60–2.88)	1.64	(1.22–2.21)
		13	Underweight	6,101 (4.6)	14 (0.2)	0.68	(0.40–1.15)	0.72	(0.42–1.22)
			Normal weight	116,349 (88.6)	447 (0.4)	1 (ref)		1 (ref)	
			Overweight	8,927 (6.8)	64 (0.7)	2.46	(1.89–3.20)	1.83	(1.40–2.39)

CI, confidence interval; CKD, chronic kidney disease; ESKD, end-stage kidney disease; IRR, incidence rate ratio; T2D, type 2 diabetes.

*Percentage of all individuals in each weight status group.

^†^Percentage of cases by weight status group.

## Discussion

Men and women with childhood BMI trajectories above average had higher rates of CKD compared to individuals with the average childhood BMI trajectory, even when including the development and duration of adult T2D in the analyses. Although similar associations were observed for ESKD, when including T2D, these were only statistically significant among women but not among men. Additionally, overweight at individual childhood ages was associated with higher rates of CKD and ESKD in comparison to childhood normal weight. When T2D was included in these analyses, associations with ESKD were only statistically significant among women.

The BMI trajectories we used have the advantage of being able to account for dynamic changes in body size across childhood. We identified 5 sex-specific BMI trajectories, which align with the contemporary United States CDC 2000 growth reference [21], and we found that CKD rates are elevated among children with BMI growth patterns below levels classified as child overweight and obesity by this reference. A limited number of studies have investigated associations between patterns of BMI growth in early life and later CKD [13,15,16]. Using the 1946 British Birth cohort, BMI trajectories created from categories of overweight (yes/no) at ages 2 to 20 years were evaluated in relation to CKD [13]. The results showed that trajectories of consistent overweight or trajectories of developing overweight were positively related to CKD risks in midlife (60 to 64 years) [13]. Although the trajectories investigated in our study are not directly comparable with those in the British study due to differences in ages examined and how the trajectories were created, both studies consistently found that patterns of early life BMI development that include overweight are important in relation to CKD risk. Nevertheless, the findings in the previous study were limited by the low number of cases (<100). Two recent studies using data from China and Australia, respectively, found that trajectories with high BMI values from child (6 to 15 or 7 to 15 years) to adult life (36 to 45 or 36 to 50 years) were positively associated with risks of subclinical renal damage in midlife used as a proxy for CKD [15,16]. These associations remained after diabetes was accounted for (either through adjustment or exclusion). Nonetheless, these studies were limited by low case numbers (257 and 82, respectively) and are not directly comparable with our study due to the age ranges investigated and as they included a subclinical outcome.

Two studies investigated if categories of body size measured at conscription examinations in late adolescence or early adulthood was associated with ESKD [12,14]. An Israeli study of male and female conscripts found that individuals with overweight and obesity at age 17 years had higher ESKD risks compared to those with normal weight, whereas no associations were found for underweight [12]. In a study of Swedish male conscripts, young adult overweight and obesity at ages 18 to 19 years was similarly associated with an increased ESKD risk compared with normal weight, whereas underweight was not [14]. Although the ages at BMI assessment differed from our study and the potential role of T2D was not evaluated, the findings from these 2 previous studies are comparable with ours and emphasize that overweight and obesity at single ages early in life may be markers of ESKD disease processes, whereas underweight most likely is not.

When including T2D as a time-varying covariate, which accounts for the time exposed to T2D before a CKD diagnosis, the associations between childhood exposures and CKD were strong and statistically significant. For ESKD, when T2D was included as a time-varying covariate, the associations with childhood BMI trajectories in women were strong and significant. In men, however, the associations were attenuated. As T2D is likely a mediator in the associations between childhood body size and adult-onset CKD, thus when including T2D in the analyses, we are not estimating the total effect of childhood body size. The impact of T2D on the development of CKD is further evident from the 10-year cumulative incidences, whereby men and women with T2D across all childhood BMI trajectories had higher cumulative incidences of CKD than individuals without T2D.

The biological mechanisms underlying the associations between excess adiposity and CKD are likely multifactorial and complex but are not fully understood. The main factors suggested to explain these links include the alterations of renal or systemic hemodynamics (including hypertension), adverse metabolic effects such as insulin resistance and chronic inflammation, and renal lipid accumulation, all contributing to kidney damage [7,9]. Another potential explanation is the tracking of body size, as it has been shown that excess adiposity at childhood ages is likely to track into adult ages [28]. Nonetheless, tracking of body size is not likely to entirely explain the association as children with severe obesity already display kidney abnormalities [7]. Additionally, a large mendelian randomization study found evidence for a causal relationship between adult BMI levels of overweight or obesity with CKD [8], supporting that the associations in our study reflect an effect of BMI.

Among the major strengths of the present study are the repeated measurements of weight and height in a large cohort of school-aged children, whereby we were able to evaluate associations with both body size at single time points as well as BMI development during childhood. Thus, we were able to take full advantage of the richness of this unique data resource by including all available childhood BMI values as opposed to restricting to information at specific ages. Selection into the cohort is limited as health examinations were performed at both public and private schools. The risk of recall bias related to memory of child body size is eliminated as the measurements were recorded as they were taken. Due to a long follow-up period with virtually no loss to follow-up as well as obtaining information on diagnoses in national and validated health registers in a nation with universal healthcare access, an adequate number of unselected cases were included. Furthermore, the risk of reverse causality affecting our associations through the inclusion of undiagnosed kidney diseases is minimized as we used a longitudinal study design with a period of minimum 15 years between the exposures and outcomes. Finally, although our cohort is historical, whereby the comparability with more contemporary populations can be questioned, historical data resources are a prerequisite to obtain a long follow-up period when examining long-term health outcomes and our study also included more recent birth cohorts.

Our study also has some limitations. Our follow-up period spans several decades; thus, it is likely that diagnostic changes have occurred as well as changes in the awareness of CKD by healthcare providers. We did not have a high and systematic coverage of laboratory tests available during the follow-up period [29]; thus, we were unable to include estimated glomerular filtration rate or albuminuria levels in the case ascertainment as recommended by the Kidney Disease Improving Global Outcomes guidelines [3]. In Denmark, patients with either mild or moderate CKD are treated in primary care, whereas patients seen in a hospital setting represent the more severe CKD cases [30]. As such, a Danish study found, unsurprisingly, that the prevalence of CKD was markedly lower using hospital-based (775 per 100,000 person-years) versus different laboratory-based (4,637 to 8,327 per 100,000 person-years) diagnoses of CKD, whereas the 1-year mortality risk was profoundly higher among individuals with hospital-based (22%) than laboratory-based CKD diagnoses (7% to 9%) [30]. Reflecting this, the median age at ESKD diagnosis was lower than the median age at a CKD diagnosis in our study. Thus, our findings are primarily generalizable to more severe cases of CKD. A small Danish validation study found that diagnoses of moderate to severe kidney disease in the Danish National Patient Register had a positive predictive value of 100% [31], suggesting that the diagnoses included in the present study are of high validity. The definition and assessment of T2D has changed across time. Whereas older cohorts only captured more advanced hospital-diagnosed cases, milder T2D cases are also registered in more recent decades, and due to the age structure of our cohort, this is when most patients with T2D in our cohort are registered. Further, we were limited by not being able to take T2D treatment regimens into account and nor did we perform formal mediation analyses of T2D on the associations between childhood body size and adult-onset CKD. To do this would have required information on potential confounders of the T2D and CKD association, and, unfortunately, this information was not systematically available for our study. Due to the nature of the available national health registers, the quality of information on hypertension (either from diagnostic codes or prescriptions) was estimated to be too poor due to its lack of specificity to evaluate its effects on the investigated associations. The identified childhood BMI trajectories rely in part on a subjective evaluation of the optimal model. However, as multiple fit indices were carefully examined, it is likely that the child BMI development and variation is accurately captured by the 5 identified trajectories for each sex, and this is also supported by the similarities with the CDC growth reference. In addition, the uncertainty of trajectory membership was accounted for by including the posterior probabilities of belonging to the trajectories in the analyses as opposed to an all-or-nothing assignment.

The children in our study were born across 57 years of birth, spanning periods before, during, and after World War II, during which numerous significant societal and medical changes occurred, including significant improvements in the healthcare system and important socioeconomic changes. Nonetheless, it is reassuring for our findings that we did not observe any birth cohort effects on these. The children included in our study were mainly of Northern European Caucasian descent, as such, our findings are likely generalizable to many Western populations. However, the potential impact of race and ethnicity on the findings requires further investigation.

In conclusion, in this study, we observed that men and women with childhood BMI trajectories above average had higher rates of adult-onset CKD and ESKD in comparison with individuals with average childhood BMI trajectories. Further, overweight at individual childhood ages was positively related to CKD and ESKD rates in comparison with childhood normal weight. Our findings when including T2D suggest that childhood body size provides information about adult-onset CKD beyond the information provided by T2D. With a worldwide growing population of children with overweight and obesity, our findings highlight the importance of continued efforts to attain or maintain a healthy weight and further add to the understanding of CKD disease etiology and potential modifiable risk factors.

## Supporting information

S1 STROBE Checklist STROBE Statement—Checklist of items that should be included in reports of *cohort studies*. (PDF)

S1 Table Sources and definitions of health-related outcomes. (PDF)

S2 Table Childhood body mass index (BMI) trajectories and chronic kidney disease by birth cohort. (PDF)

S3 Table Childhood weight status and chronic kidney disease by birth cohort. (PDF)

S4 Table Childhood body mass index (BMI) trajectories and chronic kidney disease and end-stage kidney disease. (PDF)

S1 Fig Bayesian information criteria (BIC) and proportion (%) in each childhood body mass index trajectory. (PDF)

S2 Fig Relative entropy by childhood body mass index trajectory. (PDF)

S3 Fig Estimated mean childhood body mass index trajectories among children born 1950–1954. (PDF)

S4 Fig Flow chart of individuals included from the Copenhagen School Health Records Register (CSHRR). (PDF)

## Abbreviations:

BMI: body mass index
CI: confidence interval
CKD: chronic kidney disease
CSHRR: Copenhagen School Health Records Register
ESKD: end-stage kidney disease
IQR: interquartile range
IRR: incidence rate ratio
T2D: type 2 diabetes
