# Supplementary material for: Childhood body mass index trajectories and associations with adult-onset chronic kidney disease in Denmark: A population-based cohort study
Source: PLoS Med. 2022 Sep 21;19(9):e1004098. doi: 10.1371/journal.pmed.1004098 (PMC9491561; doi:10.1371/journal.pmed.1004098)
Supplement: S1 Table — (PDF) [file pmed.1004098.s002.pdf]

S1 Table. Sources and definitions of health-related outcomes.

			International classification of disease (ICD) versions*			
**Disease**	**Health registers**	**Period**	**7**	**8**	**10**	**Anatomic Therapeutic Chemical Classification**
Chronic kidney disease	The Danish National Patient Register	1977-2017	..	249.02, 250.02, 400.39, 403.99, 404.99, 574.80, 574.90, 581.99-582.02, 582.08-583.02, 583.08-583.09, 590.09, 593.20, 593.27, 608.50, 789.09, 923.90, 924.00, 943.00, 943.40, Y9509	BFJD, E10.2, E11.2, E13.2, E14.2, I12.0-I13.2, I13.9, I15.0-I15.1, KKAS, N02-N06, N08.3, N11-N12, N15-N16, N18, N19, N28.9A, Z94.0, Z99.2, ZZ4341-ZZ4343, ZZ4346-ZZ4348	..
End-stage kidney disease	The Danish National Patient Register	1977-2017	..	Y9509, 574.80, 574.90, 608.50, 923.90, 924.00, 943.00, 943.40	BFJD, KKAS, N18.5, Z94.0, Z99.2, ZZ4341-ZZ4343, ZZ4346-ZZ4348	..
Hereditary kidney disease†	The Danish National Patient Register	1977-2017	..	..	N07, Q61.1-Q61.2, Q61.9A, Q87.8A	..
Kidney cancer†	The Danish Cancer Registry	1977-2017	180.0, 180.3	..	C64	..
Type 2 diabetes‡	The Danish National Patient Register	1977-1995	..	250	E11	..
	Steno Diabetes Center Copenhagen algorithm§ including information from: The Danish National Patient Register, The Danish National Prescription Register, The National Health Service Register, The Danish Adult Diabetes Register, The Danish Eye-screening Database	1996-2016	..	..	..	..
	The Danish National Patient Register, The Danish National Prescription Register	2017	..	..	E11	A10 (≥2 prescription refills)

* ICD codes may reflect Danish adaptations. ICD version 9 codes were never used in Denmark.

† Individuals with these diseases before the start of follow-up were excluded from the analyses. If they occurred during follow-up, they were censored on.

‡ Defined as diagnoses ≥30 years.

§ Carstensen, B & Jørgensen ME. Danish Diabetes Registers: RUKS & DMreg. Steno Diabetes Center Copenhagen. 2018.
