# Supplementary material for: Childhood body mass index trajectories and associations with adult-onset chronic kidney disease in Denmark: A population-based cohort study
Source: PLoS Med. 2022 Sep 21;19(9):e1004098. doi: 10.1371/journal.pmed.1004098 (PMC9491561; doi:10.1371/journal.pmed.1004098)
Supplement: S4 Table — (PDF) [file pmed.1004098.s005.pdf]

S4 Table. Childhood body mass index (BMI) trajectories and chronic kidney disease and end-stage kidney disease. Sex-specific incidence rate ratios (IRR) and corresponding 95% confidence intervals (CI) of the associations between childhood BMI trajectories and chronic kidney disease and end-stage kidney disease, respectively, in analyses with and without adult-onset type 2 diabetes (T2D) and duration with T2D.

			Without T2D		With T2D	
Sex	Disease	BMI trajectory	IRR	95% CI	IRR	95% CI
Men	Chronic kidney disease	Below-average	0.91	(0.85-0.98)	0.93	(0.86-1.00)
		Average	1 (ref)		1 (ref)	
		Above-average	1.14	(1.07-1.22)	1.07	(1.00-1.14)
		Overweight	1.61	(1.47-1.77)	1.25	(1.14-1.38)
		Obese	2.21	(1.79-2.72)	1.39	(1.13-1.72)
	End-stage kidney disease	Below-average	0.92	(0.77-1.11)	0.94	(0.78-1.12)
		Average	1 (ref)		1 (ref)	
		Above-average	1.09	(0.93-1.29)	1.02	(0.86-1.21)
		Overweight	1.38	(1.08-1.77)	1.08	(0.84-1.39)
		Obese	2.09	(1.26-3.48)	1.38	(0.83-2.31)
Women	Chronic kidney disease	Below-average	0.93	(0.85-1.03)	0.94	(0.85-1.04)
		Average	1 (ref)		1 (ref)	
		Above-average	1.27	(1.16-1.37)	1.18	(1.08-1.28)
		Overweight	1.52	(1.37-1.70)	1.24	(1.11-1.38)
		Obese	2.52	(2.09-3.04)	1.54	(1.28-1.86)
	End-stage kidney disease	Below-average	0.73	(0.55-0.98)	0.74	(0.56-0.99)
		Average	1 (ref)		1 (ref)	
		Above-average	1.25	(1.00-1.57)	1.17	(0.93-1.46)
		Overweight	1.81	(1.38-2.38)	1.50	(1.14-1.97)
		Obese	3.18	(2.03-4.97)	1.97	(1.25-3.11)
