# Supplementary material for: Childhood body mass index trajectories and associations with adult-onset chronic kidney disease in Denmark: A population-based cohort study
Source: PLoS Med. 2022 Sep 21;19(9):e1004098. doi: 10.1371/journal.pmed.1004098 (PMC9491561; doi:10.1371/journal.pmed.1004098)
Supplement: S1 Fig — (PDF) [file pmed.1004098.s006.pdf]

**S1 Fig. Bayesian Information Criteria (BIC) and proportion (%) in each childhood body mass index trajectory.** (A) BIC and proportion among boys in models including 1 to 8 trajectories. (B) BIC and proportion among girls in models including 1 to 8 trajectories.*

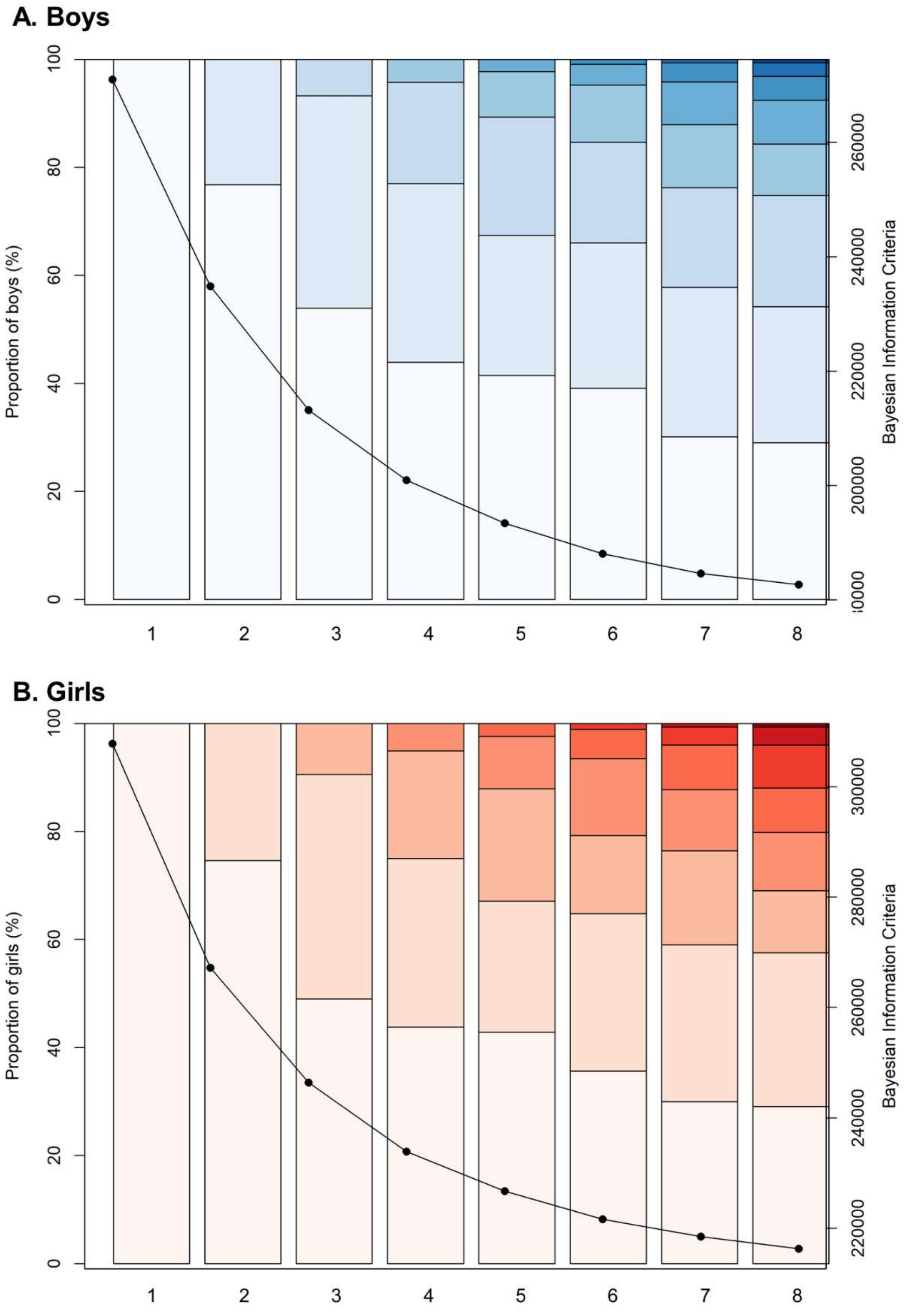


*The BIC values and the proportion of individuals in each trajectory decrease as the number of trajectories increase. The most appropriate number of trajectories is evaluated based on achieving a relatively low BIC value without the proportion in each trajectory is becoming too small. The optimal model for both sexes was evaluated as being the one that included five trajectories.
