# Supplementary material for: Childhood body mass index trajectories and associations with adult-onset chronic kidney disease in Denmark: A population-based cohort study
Source: PLoS Med. 2022 Sep 21;19(9):e1004098. doi: 10.1371/journal.pmed.1004098 (PMC9491561; doi:10.1371/journal.pmed.1004098)
Supplement: S2 Fig — (PDF) [file pmed.1004098.s007.pdf]

**S2 Fig. Relative entropy by childhood body mass index trajectory.** Relative entropy among boys (blue) and girls (red) in models including 2 to 8 trajectories.*

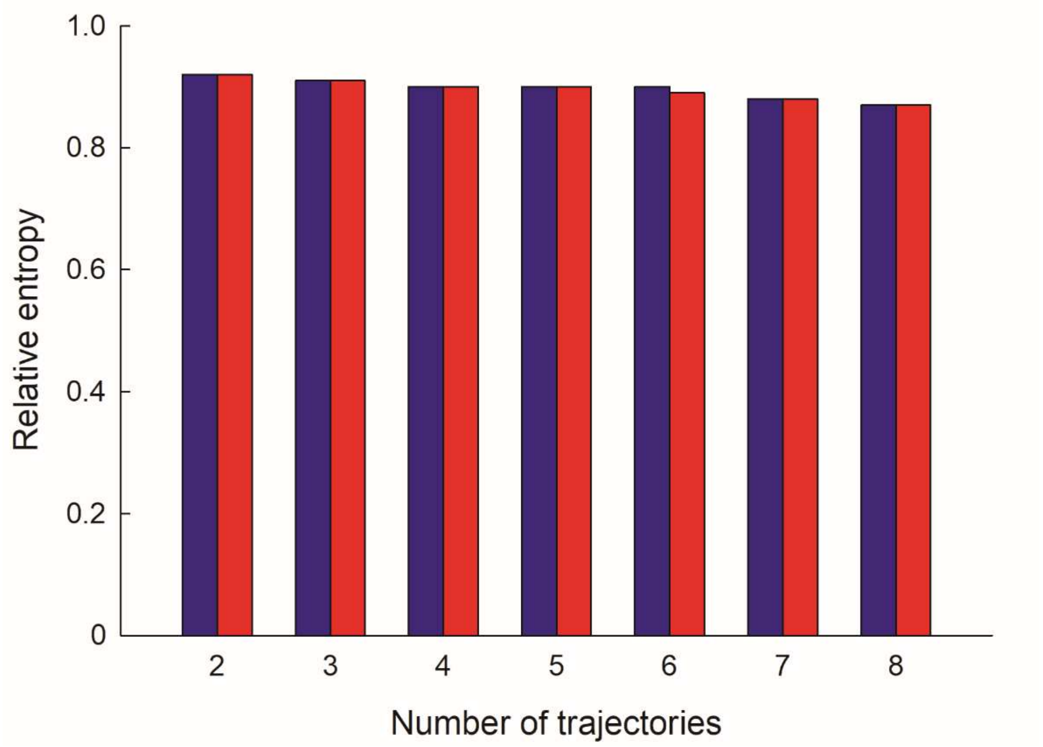


* The entropy should optimally be above 0.5.
