# Supplementary material for: Childhood body mass index trajectories and associations with adult-onset chronic kidney disease in Denmark: A population-based cohort study
Source: PLoS Med. 2022 Sep 21;19(9):e1004098. doi: 10.1371/journal.pmed.1004098 (PMC9491561; doi:10.1371/journal.pmed.1004098)

S3 Fig. Estimated mean childhood body mass index trajectories among children born 1950-1954. (A) Boys. (B) Girls.

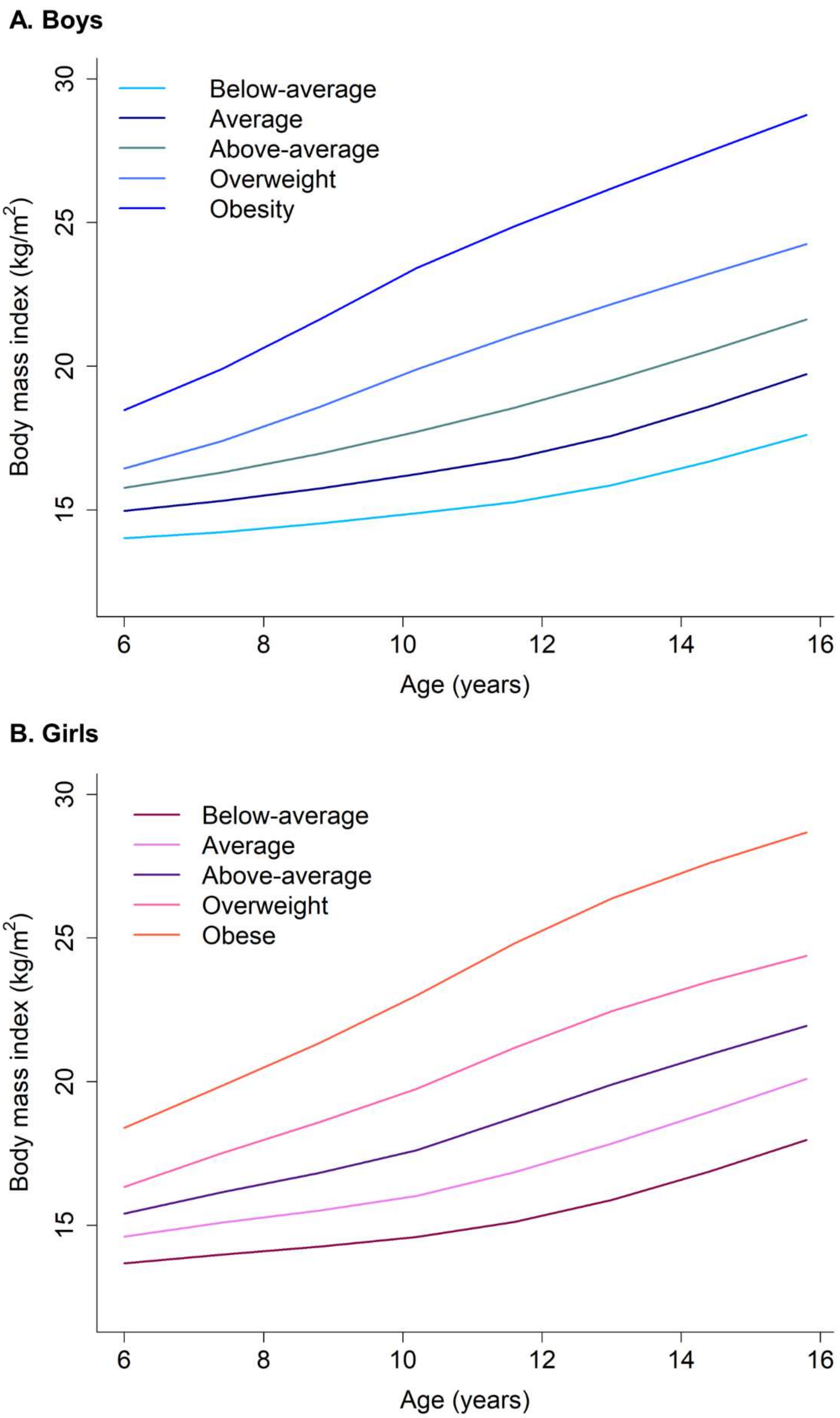

Supplement: S3 Fig — (PDF) [file pmed.1004098.s008.pdf]
