# Supplementary material for: Childhood body mass index trajectories and associations with adult-onset chronic kidney disease in Denmark: A population-based cohort study
Source: PLoS Med. 2022 Sep 21;19(9):e1004098. doi: 10.1371/journal.pmed.1004098 (PMC9491561; doi:10.1371/journal.pmed.1004098)
Supplement: S4 Fig — (PDF) [file pmed.1004098.s009.pdf]

S4 Fig. Flow chart of individuals included from the Copenhagen School Health Records Register (CSHRR).

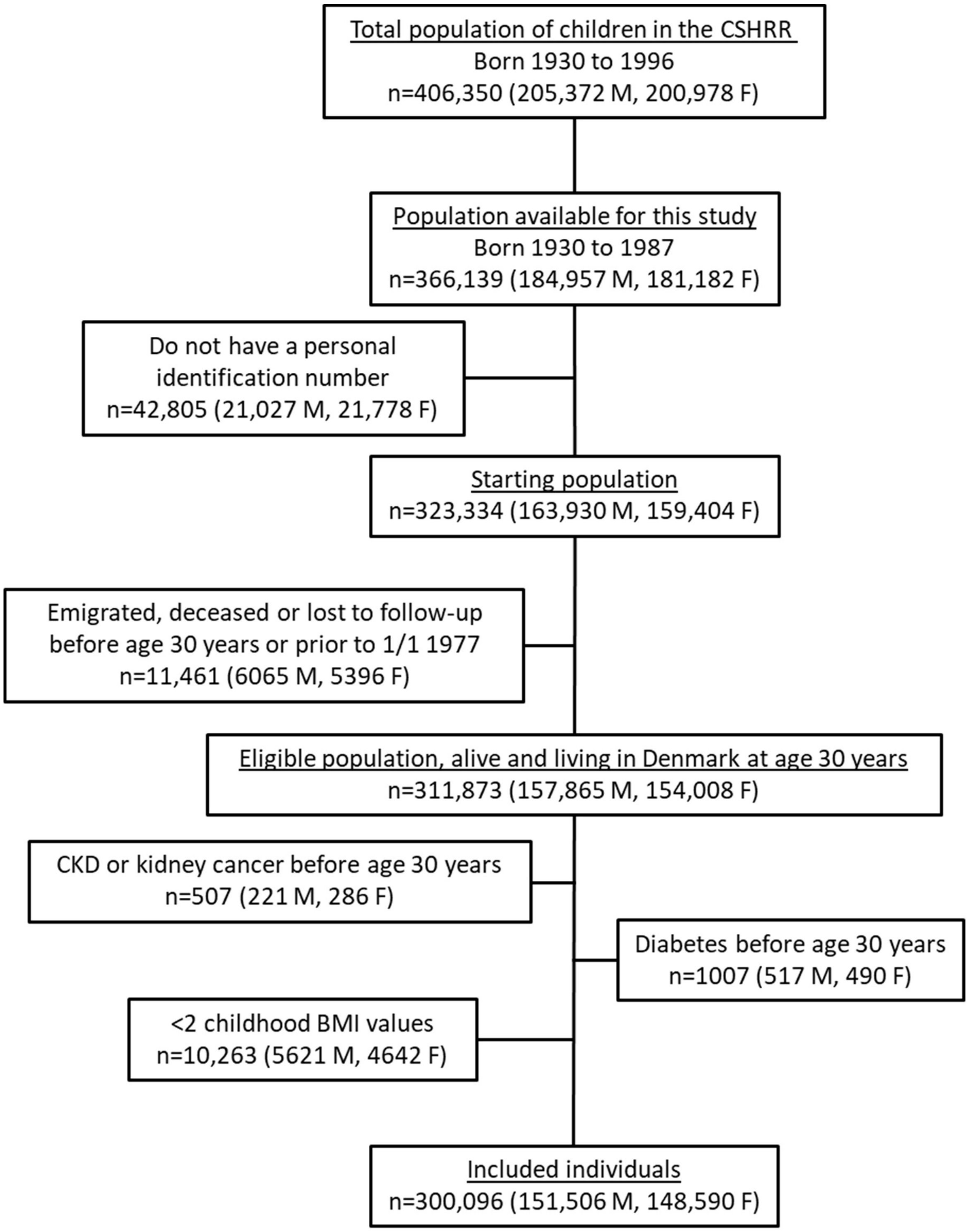

BMI, body mass index; CKD, chronic kidney disease.
