# Supplementary material for: Childhood body mass index trajectories and associations with adult-onset chronic kidney disease in Denmark: A population-based cohort study
Source: PLoS Med. 2022 Sep 21;19(9):e1004098. doi: 10.1371/journal.pmed.1004098 (PMC9491561; doi:10.1371/journal.pmed.1004098)
Supplement: S2 Table — (PDF) [file pmed.1004098.s003.pdf]

S2 Table. Childhood body mass index (BMI) trajectories and chronic kidney disease by birth cohort. Sex-specific incidence rate ratios (IRR) and corresponding 95% confidence intervals (CI) of the associations between childhood BMI trajectories and chronic kidney disease by birth cohort.

		Birth cohort						
		1930-1939		1940-1949		1950-1987		
Sex	BMI trajectory	IRR	95% CI	IRR	95% CI	IRR	95% CI	p*
Men	Below-average	0.92	(0.83-1.03)	0.89	(0.79-1.01)	0.94	(0.79-1.13)	
	Average	1.00 (ref)		1.00 (ref)		1.00 (ref)		
	Above-average	1.09	(0.98-1.21)	1.16	(1.04-1.29)	1.28	(1.09-1.52)	
	Overweight	1.34	(1.12-1.60)	1.72	(1.50-1.99)	1.87	(1.53-2.28)	
	Obese	1.43	(0.81-2.50)	2.11	(1.52-2.94)	2.91	(2.12-4.00)	0.06
Women	Below-average	0.97	(0.83-1.13)	0.86	(0.72-1.02)	1.02	(0.83-1.25)	
	Average	1.00 (ref)		1.00 (ref)		1.00 (ref)		
	Above-average	1.35	(1.20-1.53)	1.25	(1.08-1.44)	1.13	(0.94-1.37)	
	Overweight	1.55	(1.30-1.84)	1.57	(1.31-1.87)	1.48	(1.18-1.84)	
	Obese	2.70	(1.92-3.78)	2.64	(1.95-3.58)	2.23	(1.60-3.10)	0.62

* Test for interaction of birth cohort on the associations between childhood BMI trajectories and chronic kidney disease using a likelihood ratio test.
