# Supplementary material for: Childhood body mass index trajectories and associations with adult-onset chronic kidney disease in Denmark: A population-based cohort study
Source: PLoS Med. 2022 Sep 21;19(9):e1004098. doi: 10.1371/journal.pmed.1004098 (PMC9491561; doi:10.1371/journal.pmed.1004098)
Supplement: S3 Table — (PDF) [file pmed.1004098.s004.pdf]

S3 Table. Childhood weight status and chronic kidney disease by birth cohort. Sex-specific incidence rate ratios (IRR) and corresponding 95% confidence intervals (CI) of the associations between childhood weight status at ages 7, 10, and 13 years and chronic kidney disease by birth cohort.

			Birth cohort						
			1930-1939		1940-1949		1950-1987		
Sex	Age (years)	Weight status	IRR	95% CI	IRR	95% CI	IRR	95% CI	p*
Men	7	Underweight	1.04	(0.86-1.25)	1.01	(0.81-1.26)	0.95	(0.72-1.25)	
		Normal weight	1 (ref)		1 (ref)		1 (ref)		
		Overweight	1.07	(0.86-1.33)	1.44	(1.23-1.69)	1.48	(1.19-1.84)	0.15
	10	Underweight	1.14	(0.90-1.43)	0.87	(0.64-1.19)	0.95	(0.68-1.33)	
		Normal weight	1 (ref)		1 (ref)		1 (ref)		
		Overweight	1.36	(1.09-1.68)	1.83	(1.59-2.09)	1.78	(1.46-2.16)	0.09
	13	Underweight	0.95	(0.79-1.14)	0.80	(0.64-1.00)	1.01	(0.79-1.29)	
		Normal weight	1 (ref)		1 (ref)		1 (ref)		
		Overweight	1.70	(1.39-2.09)	1.90	(1.64-2.20)	2.21	(1.83-2.67)	0.22
Women	7	Underweight	1.08	(0.84-1.38)	0.98	(0.73-1.30)	0.92	(0.66-1.27)	
		Normal weight	1 (ref)		1 (ref)		1 (ref)		
		Overweight	1.47	(1.18-1.84)	1.45	(1.18-1.78)	1.44	(1.14-1.81)	0.92
	10	Underweight	0.85	(0.61-1.18)	0.76	(0.53-1.09)	0.93	(0.67-1.30)	
		Normal weight	1 (ref)		1 (ref)		1 (ref)		
		Overweight	1.67	(1.33-2.09)	1.75	(1.46-2.11)	1.36	(1.07-1.74)	0.64
	13	Underweight	0.87	(0.65-1.18)	0.73	(0.54-1.00)	0.91	(0.66-1.26)	
		Normal weight	1 (ref)		1 (ref)		1 (ref)		
		Overweight	1.93	(1.60-2.33)	1.89	(1.58-2.25)	1.81	(1.46-2.25)	0.90

* Test for interaction of birth cohort on the associations between childhood weight status and chronic kidney disease using a likelihood ratio test.
